# Inhibiting DNA methylation as a strategy to enhance adipose-derived stem cells differentiation: Focus on the role of Akt/mTOR and Wnt/β-catenin pathways on adipogenesis

**DOI:** 10.3389/fcell.2022.926180

**Published:** 2022-09-02

**Authors:** S. Ceccarelli, G. Gerini, F. Megiorni, P. Pontecorvi, E. Messina, S. Camero, E. Anastasiadou, E. Romano, M. G. Onesti, C. Napoli, C. Marchese

**Affiliations:** ^1^ Department of Experimental Medicine, Sapienza University of Rome, Rome, Italy; ^2^ Department of Maternal, Infantile and Urological Sciences, Sapienza University of Rome, Rome, Italy; ^3^ Department of Sense Organs, Sapienza University of Rome, Rome, Italy; ^4^ Department of Surgery “P. Valdoni”, Unit of Plastic Surgery “P. Valdoni”, Sapienza University of Rome, Rome, Italy; ^5^ Department of Advanced Medical and Surgical Sciences (DAMSS), University of Campania “Luigi Vanvitelli”, Naples, Italy

**Keywords:** adipose-derived adult stem cells (ASCs), epigenetic regulation, 5-azacytidine, adipogenesis, AKT/mTOR, Wnt/ beta-catenin pathway

## Abstract

Adipose-derived mesenchymal stem cells (ASCs) represent a valid therapeutic option for clinical application in several diseases, due to their ability to repair damaged tissues and to mitigate the inflammatory/immune response. A better understanding of the underlying mechanisms regulating ASC biology might represent the chance to modulate their *in vitro* characteristics and differentiation potential for regenerative medicine purposes. Herein, we investigated the effects of the demethylating agent 5-azacytidine (5-aza) on proliferation, clonogenicity, migration, adipogenic differentiation and senescence of ASCs, to identify the molecular pathways involved. Through functional assays, we observed a detrimental effect of 5-aza on ASC self-renewal capacity and migration, accompanied by actin cytoskeleton reorganization, with decreased stress fibers. Conversely, 5-aza treatment enhanced ASC adipogenic differentiation, as assessed by lipid accumulation and expression of lineage-specific markers. We analyzed the involvement of the Akt/mTOR, MAPK and Wnt/β-catenin pathways in these processes. Our results indicated impairment of Akt and ERK phosphorylation, potentially explaining the reduced cell proliferation and migration. We observed a 5-aza-mediated inhibition of the Wnt signaling pathway, this potentially explaining the pro-adipogenic effect of the drug. Finally, 5-aza treatment significantly induced ASC senescence, through upregulation of the p53/p21 axis. Our data may have important translational implications, by helping in clarifying the potential risks and advantages of using epigenetic treatment to improve ASC characteristics for cell-based clinical approaches.

## Introduction

Adipose-derived mesenchymal stem cells (ASCs) represent a population of multipotent stem cells with the ability to self-renew, which reside in the vascular stroma of adipose tissue and can differentiate into several cell types, i.e. adipocytes, osteoblasts, chondrocytes, muscle cells and vascular endothelial cells (Zuk et al., 2002). ASCs are easily obtained from subcutaneous liposuction, and they present several advantages with respect to embryonic stem cells, such as the lack of ethical issues and the lower risk for teratoma formation upon transplant (Charitos et al., 2021). The extreme differentiation plasticity and the marked migratory behavior to the site of injury makes them attractive for *in vivo* applications (Porada and Almeida-Porada, 2010). Moreover, they exert a powerful immunosuppressive effect that might both reduce the inflammatory response of the milieu and inhibit activation of immune cells, thus prolonging the survival of transplanted cells (Melief et al., 2013; Valencia et al., 2016), which makes them a feasible option for clinical application in chronic inflammatory and autoimmune diseases (De Miguel et al., 2012; Scuderi et al., 2013; Onesti et al., 2016). To date, a consistent number of clinical trials support the use of systemically administered ASCs, as cellular therapy to repair damaged tissues and to modulate the inflammatory/immune response in a wide panel of pathologies, such as cardiovascular diseases, graft versus host disease (GvHD), diabetes mellitus, osteoarthritis and inflammatory bowel disease (IBD) (Ceccarelli et al., 2020).

The multipotential differentiation of ASCs, and in particular their adipogenic commitment, is also of paramount importance for their applications in regenerative therapies. For instance, ASCs are implemented for soft tissue reconstruction in clinical conditions characterized by a large tissue void within the subcutaneous fat layer of the skin caused by traumatic injury, tumor resection, or congenital abnormalities (Shingyochi et al., 2015; Dai et al., 2016).

So, the possibility to modulate ASC adipogenesis for therapeutic purposes is especially interesting, and any advances in the study of mechanisms regulating ASC adipogenesis will significantly contribute in boosting ASC-related therapeutic efficiency in regenerative medicine. Intriguingly, the advantages of understanding the molecular mechanisms in adipocyte differentiation are not only limited to reconstructive purposes, since the ability to selectively control ASC differentiation into this specific lineage may also help setting up targeted therapies in a multitude of fields, such as intervention in obesity and other adipogenic differentiation-related disorders (Shin et al., 2020).

The ASC multilineage differentiation potential entails a delicate balance between stem cell self-renewal and differentiation. Several intracellular signaling pathways are involved in this process, such as the Wnt/β-catenin pathway, since some reports suggested that the activation of this signaling prevents both adipogenic and chondrogenic differentiation in human MSCs (Luo et al., 2013; de Winter and Nusse, 2021). Indeed, the regulation of proliferation, survival and differentiation of ASCs also occurs via the PI3K/Akt/mTOR pathway, which seems to play an important role in adipogenesis, through activation of the transcription factors C/EBPα and peroxisome proliferator–activated receptor γ (PPARγ). The latter promotes the expression of adipocyte terminal differentiation-related genes and the regulation of cell cycle, through the stimulation of D and E cyclins (Chen et al., 2013).

Furthermore, the ASC multilineage differentiation process involves changes in morphology and cell function that are determined by different patterns of gene expression (Liu et al., 2007). The implementation of these gene expression programs is regulated by epigenetic mechanisms, such as histone modification and DNA methylation, which can modify chromatin structure, thereby affecting the accessibility of target sites to regulatory proteins and modifying the affinity of transcriptional regulators for their targets (Meshorer and Misteli, 2006).

DNA methylation, catalyzed by DNA methyltransferases (DNMTs), involves the addition of a methyl group to cytosine bases and is generally associated with transcriptional silencing and chromatin condensation (Jones and Takai, 2001). In particular, maintenance of the methylation status upon DNA replication is achieved by DNMT1, which recognizes hemi-methylated DNA and methylates the daughter strand. 5-Azacytidine (5-aza) is a cytidine analog that can be incorporated into DNA of cultured cells, this causing the irreversible binding of DNMT1 to DNA and the impairment of its activity, with a subsequent global loss of methylation.

Indeed, epigenetic modifications, and in particular DNA methylation, are essential in the regulation of stem cell proliferative activity and differentiation potential (Berdasco and Esteller, 2011; Takada et al., 2014). DNA demethylation by the application of DNMT inhibitors (i.e., 5-aza) has been proved to exert beneficial effects in adult stem cell lineage commitments, such as osteogenesis, myogenesis, hepatic and cardiac differentiation (Zhou et al., 2009; Berdasco et al., 2012; Yan et al., 2014). Recent findings have provided insight that treatment of ASCs with DNA demethylating agents allows for more stable immunosuppressive properties independently of passage number, this improving their therapeutic applications (Teklemariam et al., 2014). Epigenetic modifications of the genome, such as DNA and histone acetylation/methylation, are also recognized as key factors initiating ASC aging and senescence (Muñoz-Najar and Sedivy, 2011; Pepin et al., 2020). A globally decreased DNA methylation profile has been shown to improve self-renewal ability of ASCs, to maintain their multipotency and to reduce replicative cellular senescence processes associated with prolonged *in vitro* cultures (Kornicka et al., 2017). In contrast, several reports indicate 5-aza treatment as a known senescence-inducing stimulus for cancer cells, through activation of p53 pathway or regulation of telomerase activity (Grandjenette et al., 2014; Putri et al., 2017), thus suggesting its use in cancer therapy for acute myelogenous leukemia or solid tumors (Christman, 2002). However, much remains to be discovered on how DNA methylation mechanism fully defines ASC biology. Since previous studies observed that changes in stem cell functions related to aging can drastically impair their clinical efficacy (Beane et al., 2014), the senescence onset process needs to be carefully considered when elaborating protocols for ASC personalized cellular therapy. So, a deep analysis of the mechanisms regulating ASC senescence is needed to improve expansion protocols and to identify potential strategies to delay this process, both *in vitro* and *in vivo*.

Our research focused on testing the possible ameliorating effects of epigenetically modified ASCs. Given the role of epigenetic mechanisms in the regulation of gene expression and differentiation potential (Kim et al., 2011), we investigated the effects of demethylation by 5-aza on proliferation, migration and adipogenic differentiation of human ASCs from a group of healthy donors, trying to identify the molecular pathways involved in these processes. Taking into consideration the negative impact of aging on ASC therapeutic efficacy in clinical applications, we also analyzed the potential role of 5-aza treatment in regulating *in vitro* senescence of ASCs.

## Materials and methods

### Ethics approval and consent to participate

The use of clinical samples of adipose tissue for ASC isolation complied with the Declaration of Helsinki 1975, revised in 2008, and the study methodologies have been approved by the Institutional Review Board of the Department of Experimental Medicine of the Sapienza University of Rome. Written consent was obtained from all subjects.

### Adipose-derived stem cells (ASCs) isolation and culture

Liposuction aspirates of eight healthy donors (age range 45–63 years; BMI <30 kg/m^2^) who underwent elective plastic surgery were transferred to the laboratory and processed under sterile conditions within 24 h. Isolation of ASCs was performed as previously described (Ceccarelli et al., 2018). Briefly, liposuction aspirates were washed extensively with sterile phosphate-buffered saline (PBS; Aurogene, Rome, Italy) containing 2% penicillin/streptomycin and minced. The extracellular matrix was digested with 0.075% collagenase Type I (Gibco, Paisley, UK) for 30–60 min at 37°C and 5% CO2. The suspension was filtered to remove debris and centrifuged for 5 min at 2,000 rpm. The pellets of stromal vascular fraction (SVF) containing ASCs were washed with PBS, then resuspended in the culture medium and transferred to a T75 culture flask coated with collagen Type IV (Sigma-Aldrich, St. Louis, MO, United States). ASCs were self-selected out of the SVF since they were adherent to the plastic tissue cultureware. ASCs were cultured in DMEM-Ham’s F-12 medium (vol/vol, 1:1) (DMEM/F12; Gibco) supplemented with 10% FBS, 100 U/mL penicillin, 100 mg/ml streptomycin, and 2 mM l-glutamine, and maintained in a 5% CO2 incubator at 37°C in a humidified atmosphere, with medium change twice a week. When reaching 80–90% confluence, cells were detached with 0.5 mM EDTA/0.05% trypsin (Euroclone, Milan, Italy) for 5 min at 37°C and then replated. ASCs were expanded and cell viability was assessed by using the trypan blue exclusion assay. Cell morphology was evaluated by phase contrast microscopy. Experiments were conducted between passage numbers 3 and 6, unless otherwise specified. Absence of *mycoplasma* contamination was confirmed by PCR with specific primers.

### Cell characterization and apoptosis analysis by flow cytometry

Cells at passage three were subjected to flow cytometric analyses by using a FACSCalibur cytometer (BD Biosciences, San Jose, CA, United States), as previously described (Scuderi et al., 2013; Ceccarelli et al., 2018). Briefly, cells were harvested, centrifuged, and fixed for 30 min in ice-cold 2% paraformaldehyde. The single-cell suspensions were washed in flow cytometry buffer containing PBS, 2% FBS and 0.2% Tween 20, then incubated for 30 min with the following monoclonal antibodies, conjugated to fluorescein isothiocyanate, phycoerythrin, or phycoerythrin-Cy5 (BD Biosciences): PE-Cy5 Mouse Anti-Human CD29 (Cat. No. 559882), PE Mouse Anti-Human CD34 (Cat. No. 555822), FITC Mouse Anti-Human CD44 (Cat. No. 560977), FITC Mouse Anti-Human CD45 (Cat. No. 561865), PE-Cy5 Mouse Anti-Human CD90 (Cat. No. 561972), and PE Mouse Anti-Human CD166 (Cat. No. 560903). All monoclonal antibodies were of the IgG1 isotype. Nonspecific fluorescence was determined by incubating the cells with conjugated mAb anti-human IgG1 (DakoCytomation, Glostrup, Denmark). Apoptosis was analyzed by using Annexin A5 FITC/7-AAD Kit (Beckman Coulter), following the manufacturer’s instructions. Briefly, approximately 2 × 10^5^ cells were stained with Annexin A5 FITC and 7-Amino-Actinomycin (7-AAD) for 15 min at RT in the dark. Fluorescence intensities were collected with a CytoFLEX flow cytometer (Beckman Coulter, Germany). Quadrant analysis was performed using the Kaluza software (Beckman Coulter) to quantify viable cells (Annexin A5-negative/7-AAD-negative), early apoptotic cells (Annexin A5-positive/7-AAD-negative), and late apoptotic cells (Annexin A5-positive/7-AAD-positive).

### Cell treatments

The DNA methyltransferase inhibitor 5-Azacytidine (5-aza) was purchased from Sigma-Aldrich and was reconstituted at 10 mM using dimethyl sulfoxide (DMSO; Sigma-Aldrich). For 5-aza pretreatment, the medium was changed to a freshly made culture medium containing 10 µM 5-aza. After 24 h, the 5-aza containing culture medium was refreshed for additional 24 h (total treatment 48 h). DMSO alone was used as control at 0.1% (v/v) concentration. Cells pretreated with 5-aza or DMSO were cultured in standard medium for 72 h and then subjected to analyses, unless otherwise specified. When indicated, 5-aza pretreatment was performed in the presence of the mTOR inhibitor rapamycin (0.5 mM; Sigma-Aldrich) or of human recombinant Wnt3a (100 ng/ml; R&D Systems, Minneapolis, MN, United States).

### Immunofluorescence (IF) analysis

IF was performed as previously described (Ceccarelli et al., 2014). Briefly, for ASC phenotypical characterization, cells grown on coverslips onto 24-well plates were fixed in 4% paraformaldehyde for 30 min at room temperature, followed by treatment with 0.1 M glycine (Sigma-Aldrich) in PBS for 20 min and with 0.1% Triton X-100 (Sigma-Aldrich) in PBS for additional 5 min to allow permeabilization. Cells were then assayed for the expression of specific cluster of differentiation (CD) markers by incubation with primary antibodies to CD29 (Cat. No. 303001; 1:100 dilution; BioLegend, San Diego, CA, United States) and CD166 (Cat. No. 397802; 1:20 dilution; BioLegend). Actin cytoskeleton stress fibers and focal adhesions were visualized by incubation with TRITC-Phalloidin (Cat. No. P1951; 1:100 dilution; Sigma-Aldrich) and with antibodies to vinculin (Cat. No. sc-73614; 1:100 dilution; Santa Cruz Biotechnology, Santa Cruz, CA, United States), respectively. To assess β-catenin localization and Lamin B1 expression, cells were processed as described above and incubated with antibodies to β-catenin (Cat. No. sc-6973; 1:20 dilution; Sigma-Aldrich) or lamin B1 (Cat. No. ab65986; 1:100 dilution; Abcam, Cambridge, UK). To evaluate DNA damage, cells were fixed and permeabilized as previously described and incubated with antibodies to phospho-histone H2A.X (Ser139) (γH2AX) (Cat. No. 2577; 1:100 dilution; Cell Signaling Technology, Danvers, MA, United States). To assess the expression of adipogenic markers, cells seeded on coverslips onto 24-well plates and subjected to adipogenic differentiation were fixed after 3, 7, 14 and 21 days from adipogenic induction, processed for IF as described above and then incubated with antibodies to FABP4 (Cat. No. 967799; 1:10 dilution; R&D Systems). After washing in PBS, primary antibodies were visualized using FITC-conjugated goat anti-mouse IgG (Cappel Research Products, Durham, NC, United States) or Texas Red-conjugated goat anti-rabbit IgG (Jackson ImmunoResearch Laboratories, West Grove, PA, United States). Nuclei were visualized using 4’, 6-diamidino-2-phenylindole dihydrochloride (DAPI) (Sigma-Aldrich). Nonspecific fluorescence was determined by omitting primary antibody. The single stained and merged images were acquired with a Zeiss ApoTome microscope (40x magnification) using the Axiovision software (Carl Zeiss, Jena, Germany). The percentage of FABP4-positive cells was determined by counting cells in at least six random microscopic fields for each condition.

### Growth curve analysis

Cells were seeded into 24-well plates at a density of 1 × 10^4^ cells/well and incubated at 37°C and 5% CO2 for 4 days. The number of cells was counted daily (three wells in each time per group). The mean number of cells at each counting time was plotted. The population doubling time (PDT) was calculated using the formula: PDT = T×log2/(logN1-logN2), where T is the number of days for incubation, N1 and N2 are the cell numbers determined at the beginning and end of the incubation time, respectively.

### Colony formation assay

Cells were seeded in 6-well plates in triplicate at a density of 1 × 10^3^ cells/well and incubated at 37°C and 5% CO2 for 14 days to allow colonies to grow, with medium change every 3 days. Colonies were fixed with methanol, stained with 0.1% crystal violet for 15 min at room temperature (RT) and photographed. Then, crystal violet was solubilized in 30% acetic acid in water for 15 min at RT, and absorbance was measured using the Biochrom Libra S22 UV/VIS spectrophotometer (Biochrom, Berlin, DE) at a wavelength of 595 nm. 30% acetic acid in water was used as blank control. Colony formation capacity in 5-aza-treated cells was calculated in comparison to control samples (DMSO), arbitrarily set to 1.

### Scratch test

Cells were seeded in a 6-well plate at a density of 1.0 × 10^5^ cells/well and grown until confluence, then treated with 10 µM 5-aza or DMSO for 48 h. Scratch test was performed as previously described (Ceccarelli et al., 2007). Briefly, a standardized cell-free area was introduced by scraping the confluent monolayer with a sterile tip. After intensive wash, cells were incubated for 24 h in serum-free DMEM/F12. Cells were then fixed with 4% paraformaldehyde for 30 min at room temperature. Some plates were fixed and photographed immediately after scratching representing the T0 control sample. Migration was quantitated by a measure of the % recovered scratch area after 24 h, performed using ImageJ software (v. 10.2).

### Migration assay

Cell migration was assayed with 8-µm-pore size Transwell migration chambers (Corning, Corning, NY, United States) in 24-well plates. DMEM containing 10% FBS was added in the lower chambers. Cells were trypsinized and seeded in the upper chambers at a density of 1 × 10^4^ cells/upper chamber, maintained in 200 µL of α-MEM (Sigma-Aldrich) containing 2% FBS. Cell migration was allowed to proceed for 6 h at 37°C and 5% CO2. Cells attached to the membrane in the upper chamber were gently removed with a cotton swab, whereas cells attached to the membrane in the lower chamber were fixed with ice-cold methanol for 20 min at −20°C and stained with 0.1% crystal violet for 5 min at RT. Dried membranes were cut out, mounted on glass slides and photographed. Migrating cells were counted in at least six high power fields for each experimental condition.

### Adipogenic differentiation

When reaching 100% confluence, cells were treated as described above and then incubated for 3, 7, 14 or 21 days with adipogenic differentiation medium (StemXVivo^®^ Osteogenic/Adipogenic Base Media; R&D Systems) supplemented with the respective media supplement (StemXVivo^®^ Adipogenic Supplement; R&D Systems) to induce adipogenesis.

### Quantitative real-time PCR (qRT-PCR)

ASCs were harvested and total RNA was extracted using TRIzol reagent (Invitrogen, Milan, Italy). Quantity and quality of the extracted RNA were assessed by NanoDrop (Thermo Fisher Scientific, Monza, Italy). Quantitative real-time PCR assays (qRT-PCR) were conducted in triplicate on an ABI 7500 Real Time instrument (Applied Biosystems by Life Technologies), as previously described (Nodale et al., 2014). The abundance of specific mRNAs was quantified using the following TaqMan gene expression assay probes (Applied Biosystems by Life Technologies): PPARγ (Hs01115513_m1), c/EBPα (Hs00269972_s1), sFRP-1 (Hs00610060_m1), Axin2 (Hs00610344_m1), p21 (Hs00355782_m1), p53 (Hs01034249_m1) and Bcl-2 (Hs04986394_s1). GAPDH mRNA (Hs02758991_g1) was used as endogenous control.

### Western blot (WB) analysis

Cells were lysed in RIPA buffer and processed for WB analysis as previously described (Nicolazzo et al., 2017). Briefly, total proteins (30–100 µg) were resolved under reducing conditions by 7–15% SDS-PAGE and transferred to Immobilon-FL membranes (Millipore, Billerica, MA, United States). The membranes were incubated overnight at 4 °C with primary antibodies to PPARγ (Cat. No. 2443S), phospho-Akt (Cat. No. 9271S), Akt (Cat. No. 9272S), phospho-ERK (Cat. No. 4370P), ERK2 (Cat. No. 9102), γH2AX (ser139) (Cat. No. 2577) (1:1000 dilution; Cell Signaling), phospho-mTOR (Cat. No. sc-293133), β-catenin (Cat. No. sc-7963), Cyclin D1 (Cat. No. sc-20044), p53 (Cat. No. sc-126), p21 (Cat. No. sc-6246), cleaved PARP1 (Cat. No. SC-56196) (1:200 dilution; Santa Cruz), Lamin B1 (Cat. No. ab65986; 1:4000 dilution; Abcam). Primary antibodies were followed by the appropriate horseradish peroxidase (HRP)-conjugated anti-rabbit (1:10000 dilution; Advansta, San Jose, CA, United States) and anti-mouse (1:40000 dilution; Bethyl Laboratories, Montgomery, TX, United States) secondary antibody. β-actin (Cat. No. sc-47778; 1:5000 dilution; Santa Cruz) was used as internal control. Bound antibody was detected using the WesternBright ECL HRP substrate kit (Advansta) according to the manufacturer’s instructions. Densitometric analysis was performed with Quantity One Program (Bio-Rad Laboratories S.r.l., Segrate, MI, Italy).

### Oil Red O staining

Cells were fixed in 10% formalin for 30–60 min at room temperature, incubated in 60% isopropanol for 5 min and stained with Oil Red O solution (cat. No. O0625, Sigma-Aldrich) for 5 min. The images were acquired with AxioVision software (Carl Zeiss, Jena, Germany) using a 20x objective lens. The stained oil droplets were then treated with isopropanol to elute Oil Red O dye, and the absorbance was quantified at 490 nm. Oil red O staining in 5-aza-treated cells was calculated in comparison to control samples (DMSO), arbitrarily set to 1.

### Senescence associated β-Galactosidase staining

Cellular senescence was visualized and quantified by measuring the activity of senescent-associated β-galactosidase (β-Gal) with Senescence β-Galactosidase Staining Kit (Cell Signaling), according to the manufacturer’s instructions. Briefly, cells in a 6-well plate were washed with PBS and fixed with the Fixative Solution for 15 min at room temperature, followed by staining with the β-Gal Staining Solution. Cells were incubated for 24 h at 37°C in a dry incubator and then photographed. The percentage of β-gal positive cells was calculated by counting cells in three images from each of triplicate wells for each experimental condition.

### Statistical analysis

Data were analyzed on Prism 8.0 (GraphPad Software, La Jolla, United States) and are shown as mean ± SD from three independent experiments, each conducted in triplicate, with cells obtained from at least two different donors. Two-tailed unpaired Student’s t test was used for statistical analysis. *p* values <0.05 were considered statistically significant.

## Results

### Effects of 5-aza on ASC viability, proliferation, clonogenicity and migration potential

**FIGURE 1:**
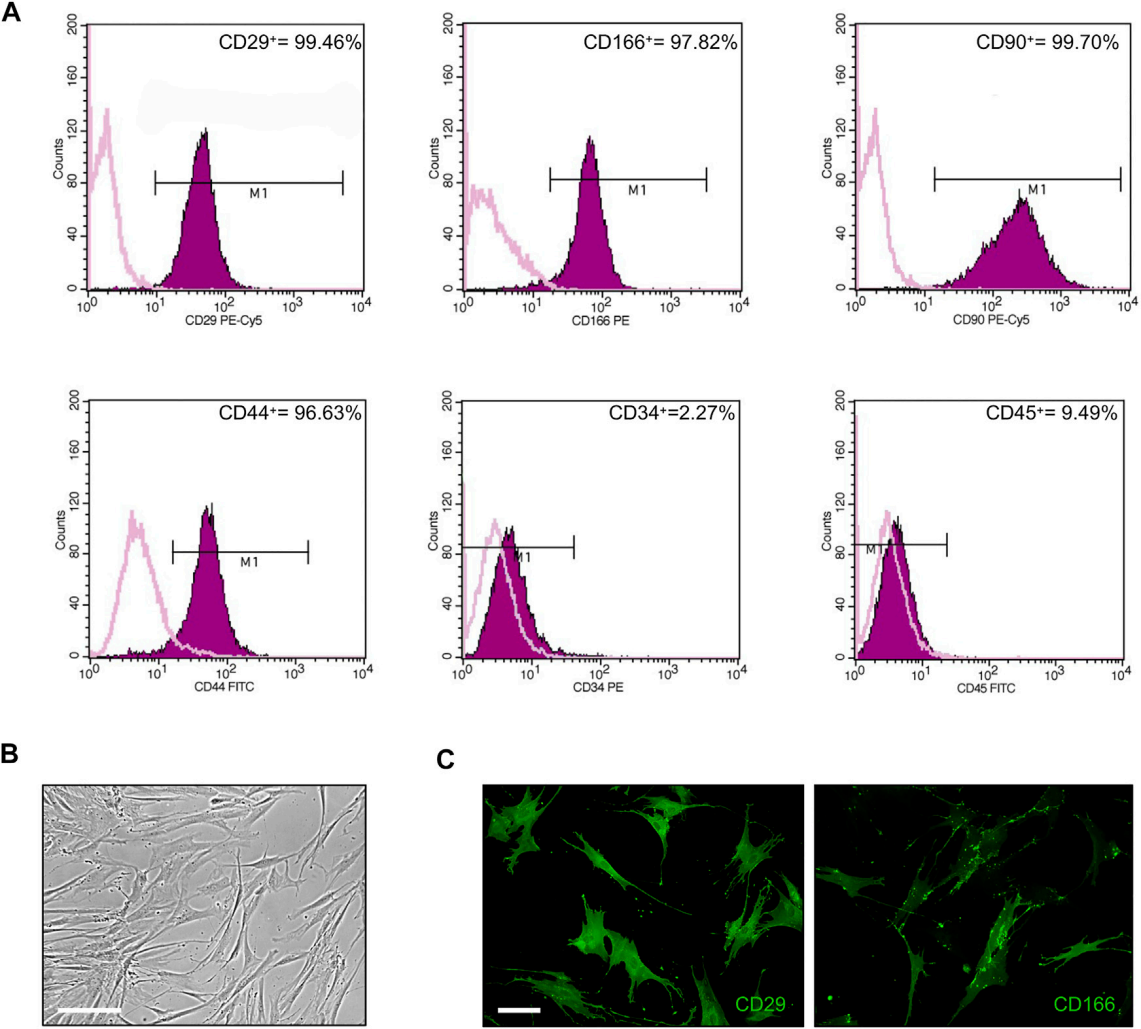
Phenotypical characterization of ASCs. **(A)** Flow cytometric analysis of ASC cells stained with monoclonal antibodies directed against the mesenchymal markers CD29, CD166, CD90, and CD44, or the hematopoietic markers CD34 and CD45. Purple areas represent patterns obtained with antibodies against the indicated markers, whereas pink lines represent the isotype-matched monoclonal antibody that served as a control. **(B)** Phase-contrast photomicrograph showing the fibroblast-like morphology of ASCs. Scale bar: 200 μm. **(C)** Representative images of CD29 and CD166 expression in ASCs by IF analysis. Scale bar: 50 μm.

First, cultured ASCs obtained from adipose tissue were phenotypically characterized by flow cytometry. FACS analysis of selected CD markers confirmed that 99.46, 97.82, 99.70 and 96.63% of ASC expressed the mesenchymal markers CD29, CD166, CD90 and CD44, respectively, while only 2.3 and 9.5% of ASC were positive for the expression of the hematopoietic markers CD34 and CD45, respectively (Figure 1A). Cultured ASCs showed the typical fibroblast-like morphology (Figure 1B). The expression of CD29 and CD166 was also confirmed by IF analysis (Figure 1C).

**FIGURE 2:**
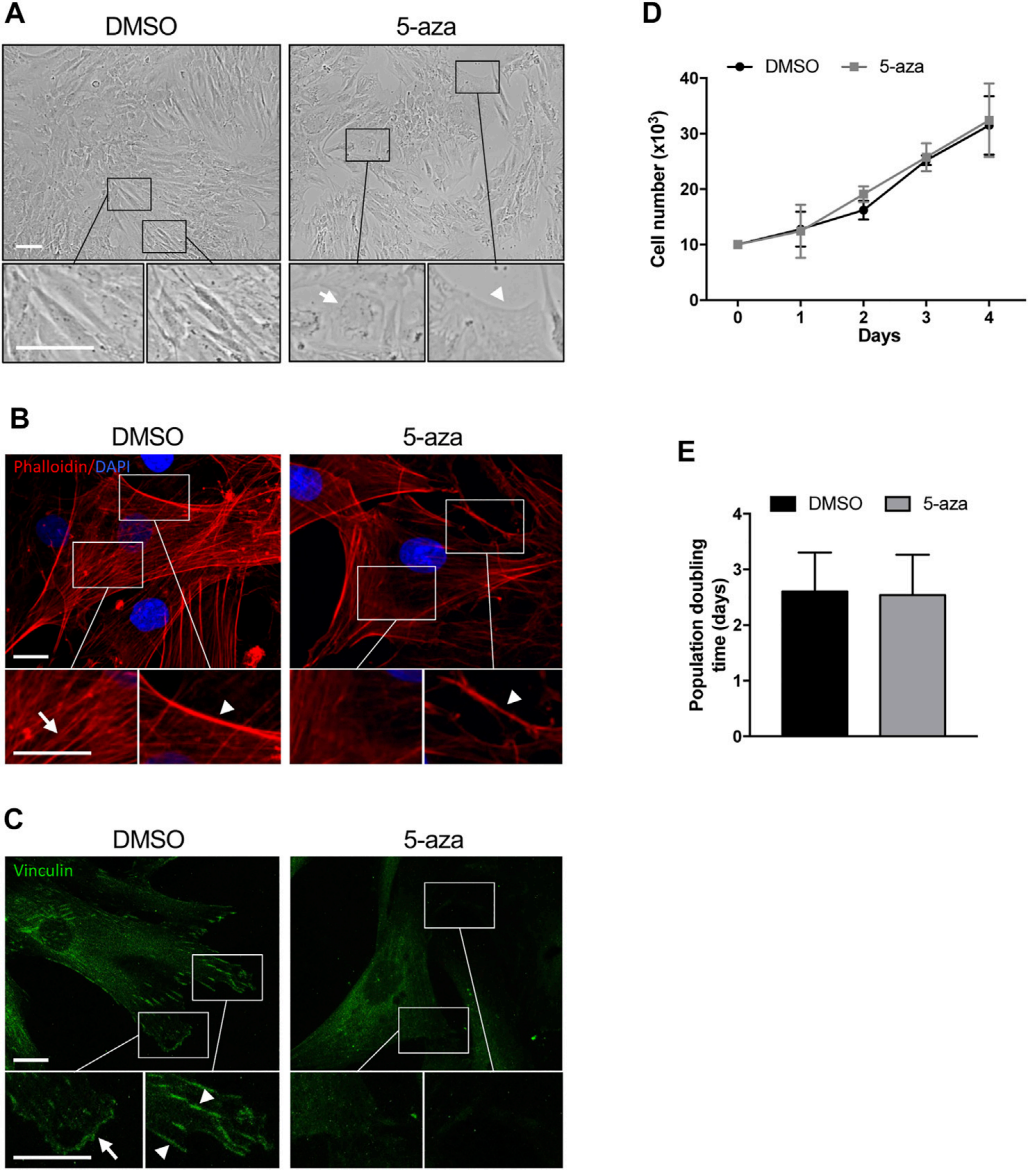
5-aza affects ASC morphology, actin cytoskeleton distribution and growth. **(A)** Phase-contrast photomicrographs showing the morphology of ASCs pretreated with 5-aza or DMSO. Representative areas of the full image are presented as enlargements in lower panels. Scale bars: 100 μm. **(B)** Fluorescence images showing phalloidin labeling of actin filaments (red) in ASCs pretreated with 5-aza or DMSO. Nuclei were visualized using 4′, 6-diamido-2-phenylindole dihydrochloride (DAPI) (blue). Representative areas of the full image are presented as enlargements in lower panels. Scale bars: 10 μm. **(C)** Fluorescence images showing Vinculin labeling of focal adhesions (green) in ASCs pretreated with 5-aza or DMSO. Representative areas of the full image are presented as enlargements in lower panels. Scale bars: 10 μm. **(D)** Growth kinetics of ASCs pretreated with 5-aza or DMSO. **(E)** Population doubling time calculated from the exponential growing phase of ASCs. Bars represent means ± SD of three independent experiments, each performed in triplicate.

In order to investigate the effects of the demethylating agent 5-aza in ASCs, cells were pretreated with 10 µM 5-aza or DMSO for 48h, and then cultured for 72 h in standard medium prior to further analyses. ASC density and morphology after pretreatment were evaluated by phase contrast microscopy. We noticed a slightly decreased confluence in ASCs pretreated with 5-aza with respect to DMSO-treated cells (Figure 2A). Both ASCs exhibited a fibroblast-like morphology, but in the 5-aza group some cells showed enlarged nuclei (arrow) and flat, irregular shape (arrowhead) (Figure 2A, enlargements). Actin cytoskeleton organization, evaluated by fluorescence staining of F-actin filaments with TRITC-Phalloidin (Figure 2B, red), revealed a consistent number of stress fibers, forming long linear bundles, which extended throughout the cell (arrow) in DMSO-treated cell (Figure 2B, enlargements). Treating ASCs with 5-aza, actin stress fibers were significantly decreased in the cell body, and their size was clearly decreased in the cortex (arrowheads) (Figure 2B, enlargements), although the morphology of the cells was not markedly altered. Immunofluorescence staining with Vinculin to visualize focal adhesions (Lehtimäki et al., 2021) (Figure 2C, green) revealed a high number of vinculin-positive focal adhesion complexes on the cell membrane (arrowheads) and in particular on the cell edges (arrow) (Figure 2C, enlargements) of DMSO-treated cells. In contrast, 5-aza-treated cells showed very few focal adhesion complexes.

Cell growth kinetics revealed no significant differences in the ASC proliferation potential after 5-aza pretreatment (Figure 2D). Moreover, 5-aza did not show significant effects on PDT (2.54 days vs*.* 2.61 days of DMSO; Figure 2E). So, this concentration of the drug seemed to show no deleterious effect on cell viability.

**FIGURE 3:**
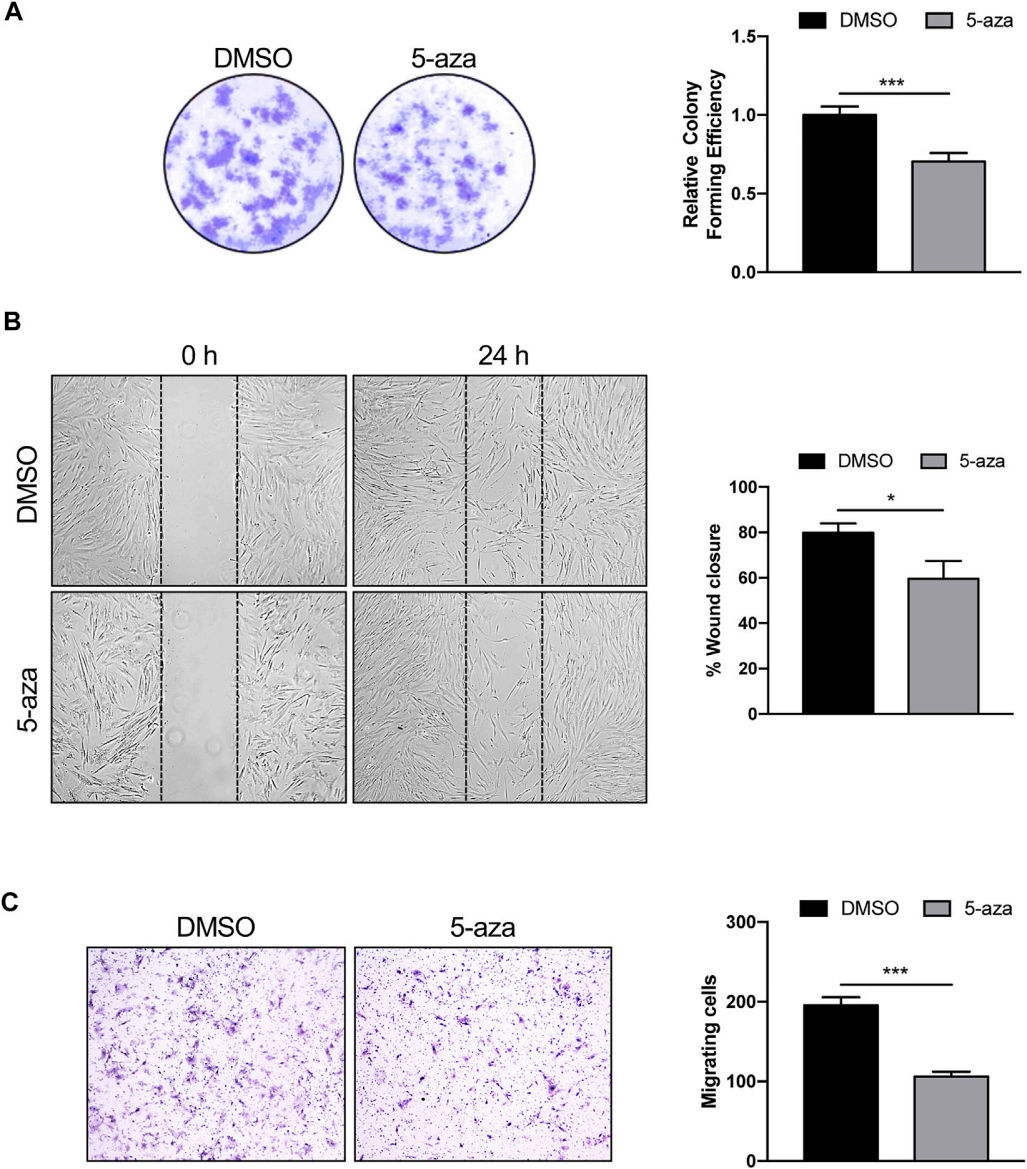
5-aza affects ASC proliferation and migration. **(A)** Colony formation assay showing the effect of 5-aza on clonogenic ability of ASCs. Colonies were stained with crystal violet. Colony forming efficiency was calculated by crystal violet absorbance. **(B)** Scratch test showing the effect of 5-aza on ASC migration. The percentage of recovered area at 24 h was measured by ImageJ software. **(C)** Transwell assay of ASCs pretreated with 5-aza or DMSO. Migrated cells were stained with crystal violet. Migration efficiency was calculated by counting the number of migrating cells. Bars represent means ± SD of three independent experiments, each performed in triplicate. *p < 0.05, ***p < 0.0005 vs*.* DMSO.

The effects of 5-aza on ASC self-renewal capacity were further evaluated through analyzing their ability to form colonies at low-density inoculation, as previously reported (Liu et al., 2017). As indicated in Figure 3A, 5-aza pretreatment significantly decreased the amount of ASC colonies, with a 30% reduction in colony forming ability in 5-aza-treated cells relative to DMSO-treated controls.

Next, scratch test and transwell assay were used to analyze the migration ability of ASCs pretreated or not with 5-aza, since migratory potential is strictly related to ASC repair ability *in vivo* (Habisch et al., 2007). The results of the scratch test showed a 25% decrease of migration ability at 24 h in cells subjected to 5-aza pretreatment compared to that in the DMSO-treated cells (Figure 3B). Furthermore, the transwell assay revealed a 46% reduction in the number of migratory cells of the 5-aza group compared to that of the DMSO group within 6 h (Figure 3C).

### Effects of 5-aza pretreatment on ASC adipogenesis

**FIGURE 4:**
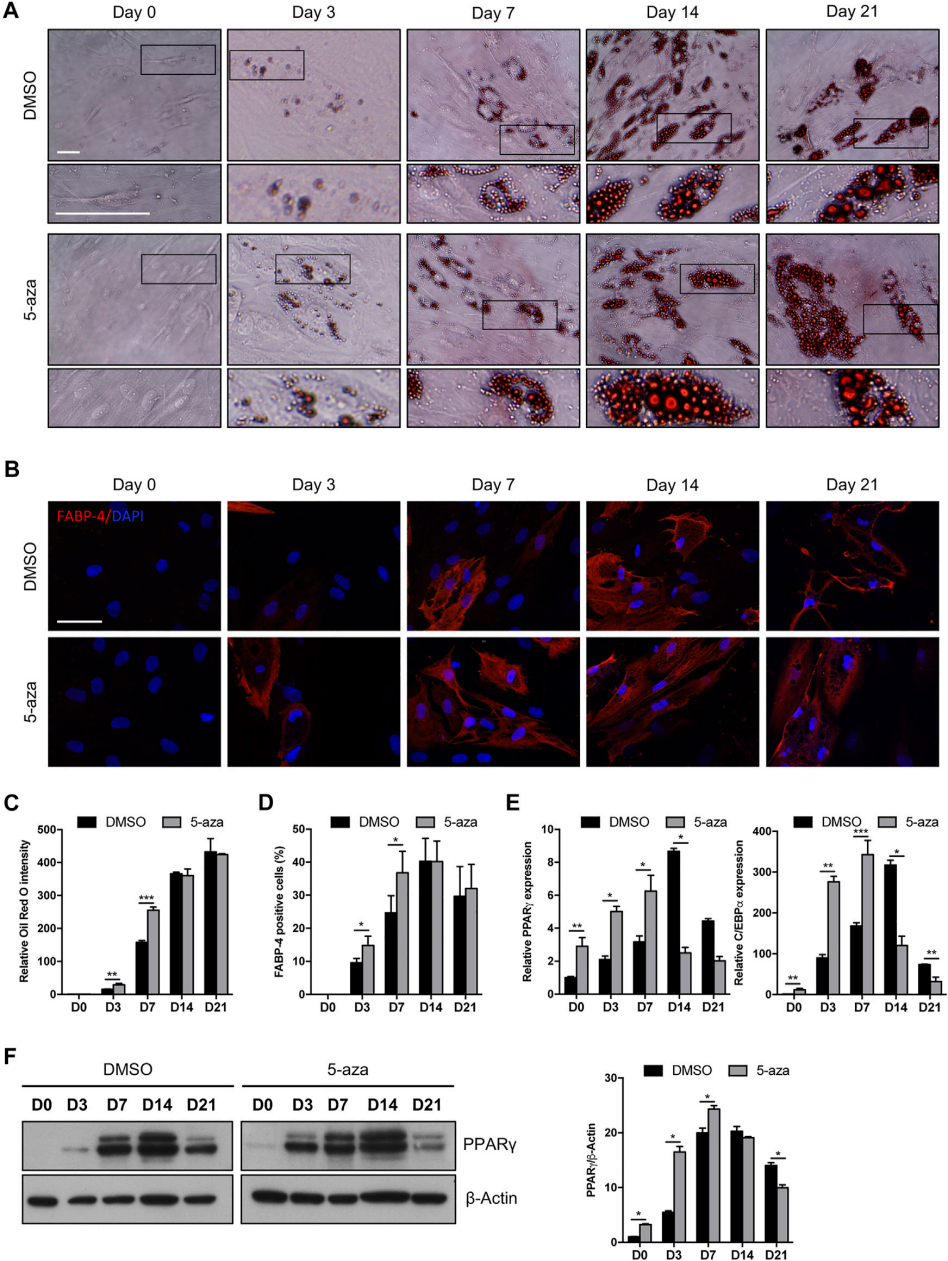
5-aza affects ASC adipogenic differentiation. **(A)** Phase-contrast photomicrographs showing ASCs pretreated with 5-aza or DMSO and stained with Oil Red O to visualize lipid accumulation at day 0, 3, 7, 14 and 21 of adipogenic induction (D0-D21). Representative areas of the full image are presented as enlargements in lower panels. Scale bars: 50 µm. **(B)** IF analysis showing the expression of FABP4 (red). Nuclei were visualized using DAPI (blue). Scale bar: 50 µm. **(C)** Quantitative analysis of lipid droplets by measuring Oil Red O absorbance. **(D)** Percentage of FABP4-positive cells, determined by counting the number of FABP4-positive cells vs. total number of cells in ten random areas for each condition. **(E)** PPARγ and c/EBPα mRNA expression, evaluated by qRT-PCR. mRNA levels were normalized to GAPDH mRNA expression. **(F)** Western blot analysis of PPARγ protein expression. β-Actin was used as internal control. Densitometric analysis was reported as relative expression with respect to D0 of DMSO-treated cells. Bars represent means ± SD of three independent experiments, each performed in triplicate. *p < 0.05, **p < 0.005, ***p < 0.0005 vs*.* DMSO.

We investigated the effects of 5-aza on lineage-specific differentiation by evaluating adipogenesis in ASC cultures pretreated or not with 10 µM 5-aza for 48 h before adipogenic induction. Cells pretreated with DMSO or 5-aza were cultured in Adipocyte Differentiation Medium for 3, 7, 14 or 21 days and differentiation was assessed by evaluation of intracellular lipid accumulation through staining with Oil Red O (Figure 4A) and by the expression of the adipocyte-specific fatty acid binding protein 4 (FABP4) through IF analysis (Figure 4B).

5-aza-treated cells showed a significantly increased accumulation of lipid droplets compared to DMSO-treated cells at Day 3 and Day 7 of induction, as determined by quantification of Oil Red-O staining (+95% and +71% vs. DMSO, respectively) (Figure 4C). The same trend was observed by counting the percentage of FABP4-positive cells at 3 and 7 days (+55% and +49% vs. DMSO, respectively) (Figure 4D). At later times of adipogenic induction, no significant differences between 5-aza- and DMSO-treated cells were observed. Interestingly, 5-aza pretreatment seemed to increase the size of lipid droplets at every time of induction (Figure 4A, insets).

We then analyzed the expression of the master regulators of adipogenesis, PPARγ and c/EBPα, in response to 5-aza pretreatment. As expected, expression of both PPARγ and c/EBPα mRNA was significantly increased in the cultures induced towards adipogenesis, starting from 3 days, with a maximum at 14 days and then a decrease at 21 days. Interestingly, 5-aza pretreatment was able to significantly increase PPARγ and c/EBPα expression at 0, 3 and 7 days of induction (PPARγ: 2.9-, 2.4- and 2.0-fold vs. DMSO, respectively; c/EBPα: 11.4-, 3.1- and 2.0-fold vs. DMSO, respectively) (Figure 4E). Moreover, 5-aza seemed also to further decrease both the differentiation markers at later times of induction (Figure 4E). PPARγ protein expression was also evaluated by WB analysis (Figure 4F), and densitometric analysis confirmed a significant increase at 0, 3 and 7 days, and a decrease at 21 days. At 14 days, despite the lower expression of PPARγ mRNA observed in 5-aza-treated cells, its protein levels still remain similar to those of DMSO-treated cells, potentially suggesting the intervention of post-transcriptional mechanisms regulating protein turnover. Overall, early ASC adipogenesis was positively affected by 5-aza pretreatment.

### 5-Aza pretreatment modulates Akt/mTOR pathway

**FIGURE 5:**
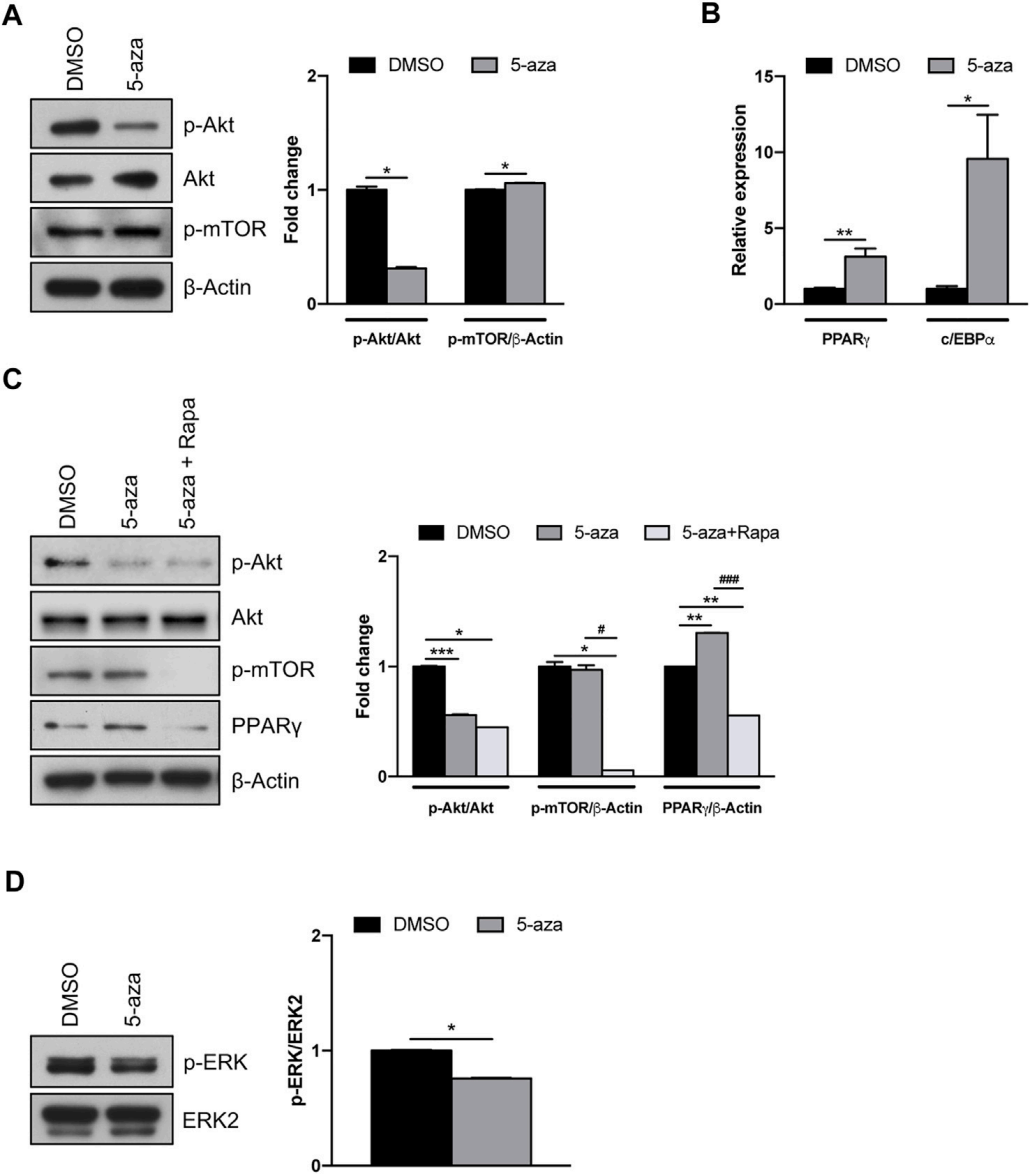
5-aza effects on Akt/mTOR and MAPK signaling pathways. **(A)** Western blot analysis of phospho-AKT, AKT and phospho-mTOR protein expression in ASCs pretreated with 5-aza or DMSO. β-Actin was used as internal control. **(B)** PPARγ and c/EBPα mRNA expression, evaluated by qRT-PCR. mRNA levels were normalized to GAPDH mRNA expression. **(C)** Western blot analysis of phospho-AKT, AKT, phospho-mTOR and PPARγ protein expression in ASCs pretreated with 5-aza or DMSO in the presence or not of rapamycin. β-Actin was used as internal control. **(D)** Western blot analysis of phospho-ERK and ERK2 protein expression. Densitometric analysis was reported as relative expression with respect to DMSO-treated cells. Bars represent means ± SD of three independent experiments, each performed in triplicate. *p < 0.05, **p < 0.005, ***p < 0.0005 vs*.* DMSO. #*p* < 0.05, ###*p* < 0.0005 vs*.* 5-aza.

Akt/mTOR signaling pathway has a pivotal role in the regulation of proliferation, migration and differentiation of mesenchymal stem cells (Chen et al., 2013). However, a direct effect of 5-aza on this pathway in ASCs has not yet been assessed. We demonstrated that 5-aza pretreatment for 48 h markedly impairs Akt phosphorylation in ASCs (0.3-fold vs. DMSO-treated cells), without affecting Akt total protein expression (Figure 5A). We then assessed the activation of the downstream protein mTOR, which is known to induce the two main adipogenic transcriptional factors PPARγ and c/EBPα (Yu et al., 2008). Interestingly, we observed that after 5-aza pretreatment mTOR phosphorylation shows a slight increase (1.1-fold vs. DMSO) (Figure 5A). We also confirmed the ability of 5-aza pretreatment to induce PPARγ and c/EBPα mRNA expression (3.1-fold and 9.6-fold vs. DMSO, respectively) (Figure 5B). An Akt-independent activation of mTOR might explain the positive effects of 5-aza pretreatment on adipogenic differentiation. To test this hypothesis, we performed 5-aza pretreatment in the presence of rapamycin, a known mTOR inhibitor. As reported in Figure 5C, PPARγ increase observed upon 5-aza treatment (1.3-fold vs. DMSO) was completely abolished by rapamycin (0.6-fold vs. DMSO), this confirming the central role of mTOR in 5-aza-mediated enhancement of adipogenic induction.

Since the MAPK pathway also plays a key role in different cellular functions, including proliferation and differentiation (Ren et al., 2019), we further investigated the effects of 5-aza on ERK activation. Our results, showing a reduction in ERK phosphorylation upon 5-aza pretreatment (0.7-fold vs. DMSO; Figure 5D), might also explain the reduced clonogenicity in 5-aza-treated cells observed in colony forming assay (Figure 3A).

### 5-Aza pretreatment inhibits Wnt/β-catenin pathway

**FIGURE 6:**
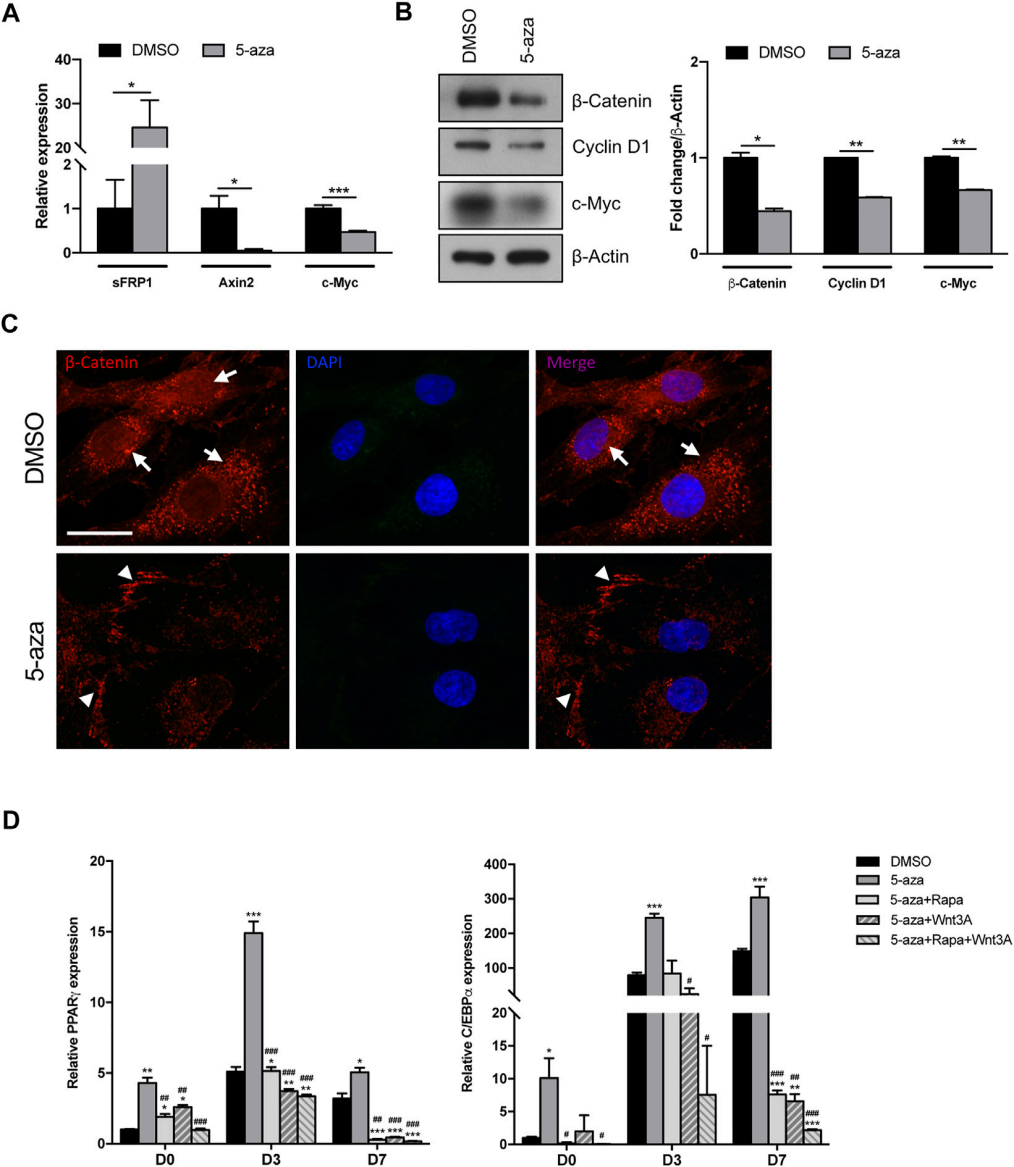
5-aza effects on Wnt/β-catenin signaling pathway. **(A)** sFRP-1 and Axin2 mRNA expression in ASCs pretreated with 5-aza or DMSO, evaluated by qRT-PCR. mRNA levels were normalized to GAPDH mRNA expression. **(B)** Western blot analysis of β-catenin, Cyclin D1 and c-Myc protein expression. β-Actin was used as internal control. Densitometric analysis was reported as relative expression with respect to DMSO-treated cells. **(C)** IF analysis showing the expression of β-catenin (red). Nuclei were visualized using 4′, 6-diamido-2-phenylindole dihydrochloride (DAPI) (blue). Scale bar: 50 µm. **(D)** PPARγ and c/EBPα mRNA expression, evaluated by qRT-PCR. mRNA levels were normalized to GAPDH mRNA expression. Bars represent means ± SD of three independent experiments, each performed in triplicate. *p < 0.05, **p < 0.005, ***p < 0.005 vs*.* DMSO; #*p* < 0.05, ##*p* < 0.005, ###*p* < 0.0005 vs*.* 5-aza.

The adipogenic commitment of ASCs is regulated by a complex and highly orchestrated gene expression program and many developmental signaling pathways (Ambele et al., 2020). To further explore the mechanisms through which 5-aza regulates adipogenic differentiation, we tested whether 5-aza pretreatment affects the endogenous expression of key adipogenic regulators belonging to the Wnt/β-catenin pathway. Intriguingly, after 5-aza pretreatment, the mRNA expression of the Wnt inhibitor sFRP-1 was dramatically upregulated (24.6-fold; Figure 6A). Consistently, 5-aza treatment also induced the downregulation of the Wnt target genes, such as Axin2, as assessed by means of qRT-PCR (0.05-fold; Figure 6A), as well as Cyclin D1 and c-Myc, as assessed by WB (0.6-fold and 0.7-fold, respectively; Figure 6B). Also, β-catenin protein expression is significantly affected (0.4-fold; Figure 6B). Moreover, IF analysis with ApoTome microscope confirmed partial β-catenin degradation and also revealed that its intracellular distribution was significantly altered by 5-aza, with an impairment of nuclear translocation and an increase of membrane signal to the detriment of cytoplasmic one (Figure 6C).

These data imply that the 5-aza ability of enhancing adipogenic differentiation in ASC cells might be exerted also by inactivating canonical Wnt signaling.

To further confirm the role of both mTOR and Wnt/β-catenin pathways in 5-aza-mediated enhancement of adipogenic differentiation, we performed adipogenic induction in cells pretreated with 5-aza alone or in the presence of the mTOR inhibitor rapamycin, of the human recombinant Wnt3A protein, a known inducer of canonical Wnt signaling pathway, or both. As expected, we observed that the increased expression of adipogenic markers PPARγ and c/EBPα induced by 5-aza at 3 and 7 days (PPARγ: 2.9- and 1.6-fold vs*.* DMSO, respectively; c/EBPα: 3.1-, and 2.0-fold vs*.* DMSO, respectively) was impaired by either rapamycin (PPARγ: 1.0- and 0.1-fold vs*.* DMSO, respectively; c/EBPα: 1.1-, and 0.1-fold vs*.* DMSO, respectively) or Wnt3A (PPARγ: 0.7- and 0.1-fold vs*.* DMSO, respectively; c/EBPα: 0.3-, and 0.04-fold vs*.* DMSO, respectively), and much more in the presence of rapamycin and Wnt3A together (PPARγ: 0.7- and 0.1-fold vs*.* DMSO, respectively; c/EBPα: 0.1-, and 0.01-fold vs*.* DMSO, respectively) (Figure 6D). These data are in line with our hypothesis of an involvement of the two pathways in mediating 5-aza effect on ASC adipogenesis.

### 5-Aza promotes ASC cell senescence

**FIGURE 7:**
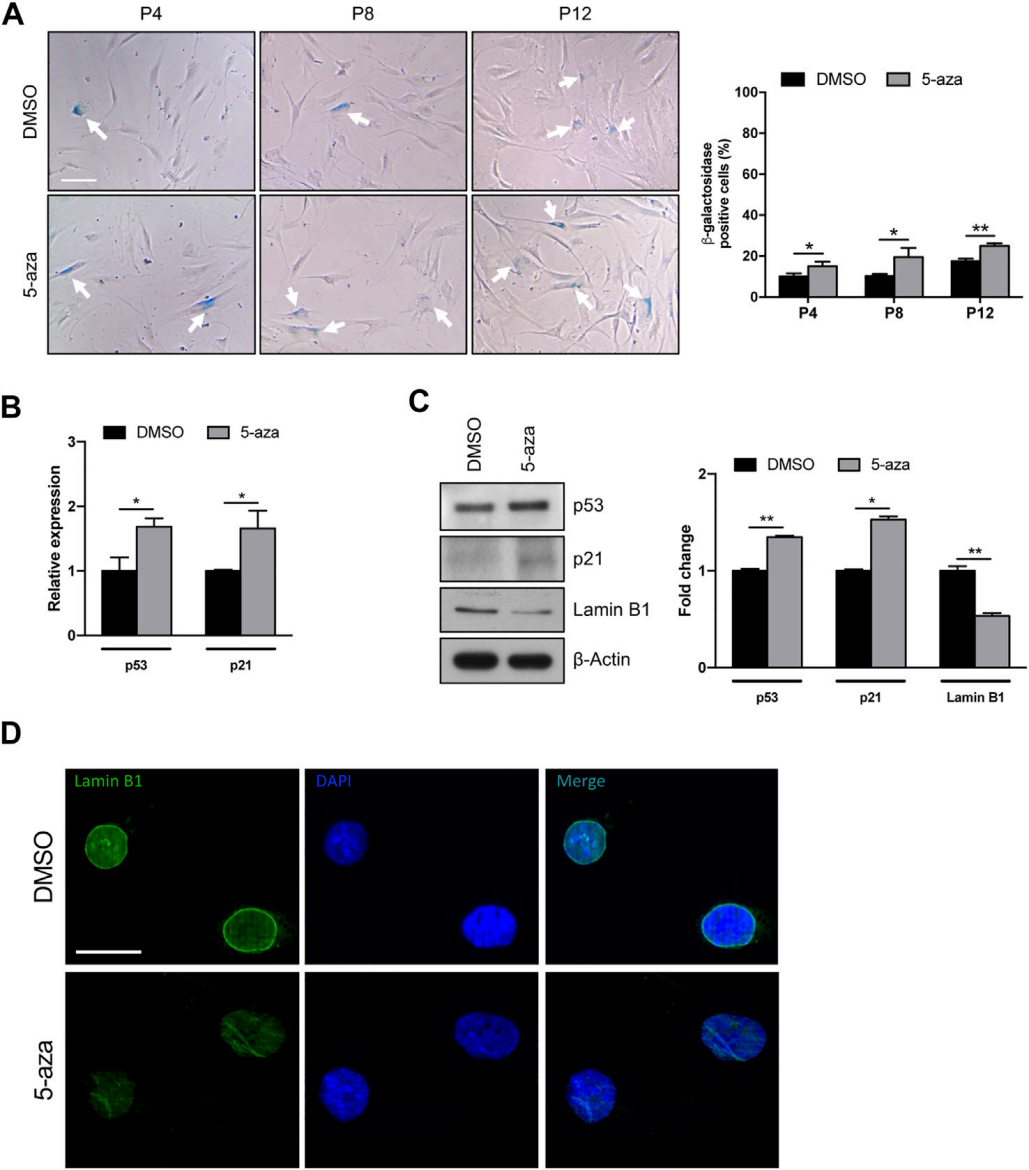
5-aza affects ASC senescence. **(A)** Phase-contrast photomicrographs showing ASCs pretreated with 5-aza or DMSO at passage 4, 8 and 12 (P4-P12), stained with β-Galactosidase Staining Kit to visualize senescent cells. The percentage of β-Gal-positive cells was determined by counting the number of blue cells vs. total number of cells in ten random areas for each condition. **(B)** P53 and p21 mRNA expression, evaluated by qRT-PCR. mRNA levels were normalized to GAPDH mRNA expression. **(C)** Western blot analysis of p53, p21 and lamin B1 protein expression. β-Actin was used as internal control. Densitometric analysis was reported as relative expression with respect to DMSO-treated cells. **(D)** IF analysis showing the expression of Lamin B1 (green). Nuclei were visualized using DAPI (blue). Scale bar: 50 µm. Bars represent means ± SD of three independent experiments, each performed in triplicate. *p < 0.05, **p < 0.005 vs*.* DMSO.

Given the potential use of 5-aza as a senescence inducer (Purcell et al., 2014), and the above results indicating flattened morphology, reduced colony formation ability and Cyclin D1 downregulation upon 5-aza pretreatment, we wondered whether 5-aza was able to induce ASC senescence. We analyzed ASCs at early, intermediate and late passages. Cells at P2 were treated with 10 µM 5-aza or DMSO for 48 h and then cultured in standard medium. At P4, P8 and P12, 5-aza- or DMSO-treated cells were stained with the Senescence β-Galactosidase Staining Kit and the percentage of senescent cells was calculated. As shown in Figure 7A, 5-aza-treated ASCs at P4, P8 and P12 showed a significant increase of senescent cells with respect to DMSO (14.9% vs. 10.2%, 19.4% vs. 10.3% and 24.9% vs. 17.6% of DMSO, respectively), suggesting that 5-aza pretreatment was able to significantly induce senescence in cultured ASCs.

We next analyzed the effects of 5-aza on senescence-related gene expression. We observed an increase of both p53 and p21 mRNA levels upon 5-aza pretreatment (1.7-fold; Figure 7B). As shown in Figure 7C, the protein expression of p53 and p21 was also significantly increased in ASCs pretreated with 5-aza compared with ASCs pretreated with DMSO (1.3-fold and 1.5-fold, respectively). We also investigated the expression of Lamin B1, a protein known to be downmodulated in senescent cells (Hernandez-Segura et al., 2018). WB experiments shown in Figure 7C demonstrated a significant downregulation of Lamin B1 protein expression upon 5-aza treatment (0.5-fold vs. DMSO), which was further confirmed by IF experiments showing an impairment of Lamin B1 nuclear membrane signal in 5-aza-treated cells (Figure 7D). Another feature of senescence is the induction of the senescence-associated secretory phenotype (SASP), which comprises cytokines and soluble factors released from senescent cells. We therefore measured the SASP components in supernatants of ASC cells treated with DMSO or 5-aza using cytokine array. It revealed significantly increased secretion of SDF-1, MIF, PAI-1, IL-6 and IL-8 (Supplementary Figure S1A). All these molecules are reported to be significantly altered between presenescent and senescent states (Coppé et al., 2010). The increase in PAI-1 and IL-6 was also confirmed by qRT-PCR analysis (2.5- and 2.4-fold, respectively; Supplementary Figure S1B). Altogether, these data indicate that 5-aza pretreatment might increase cell senescence of ASCs during *in vitro* expansion.

**FIGURE 8:**
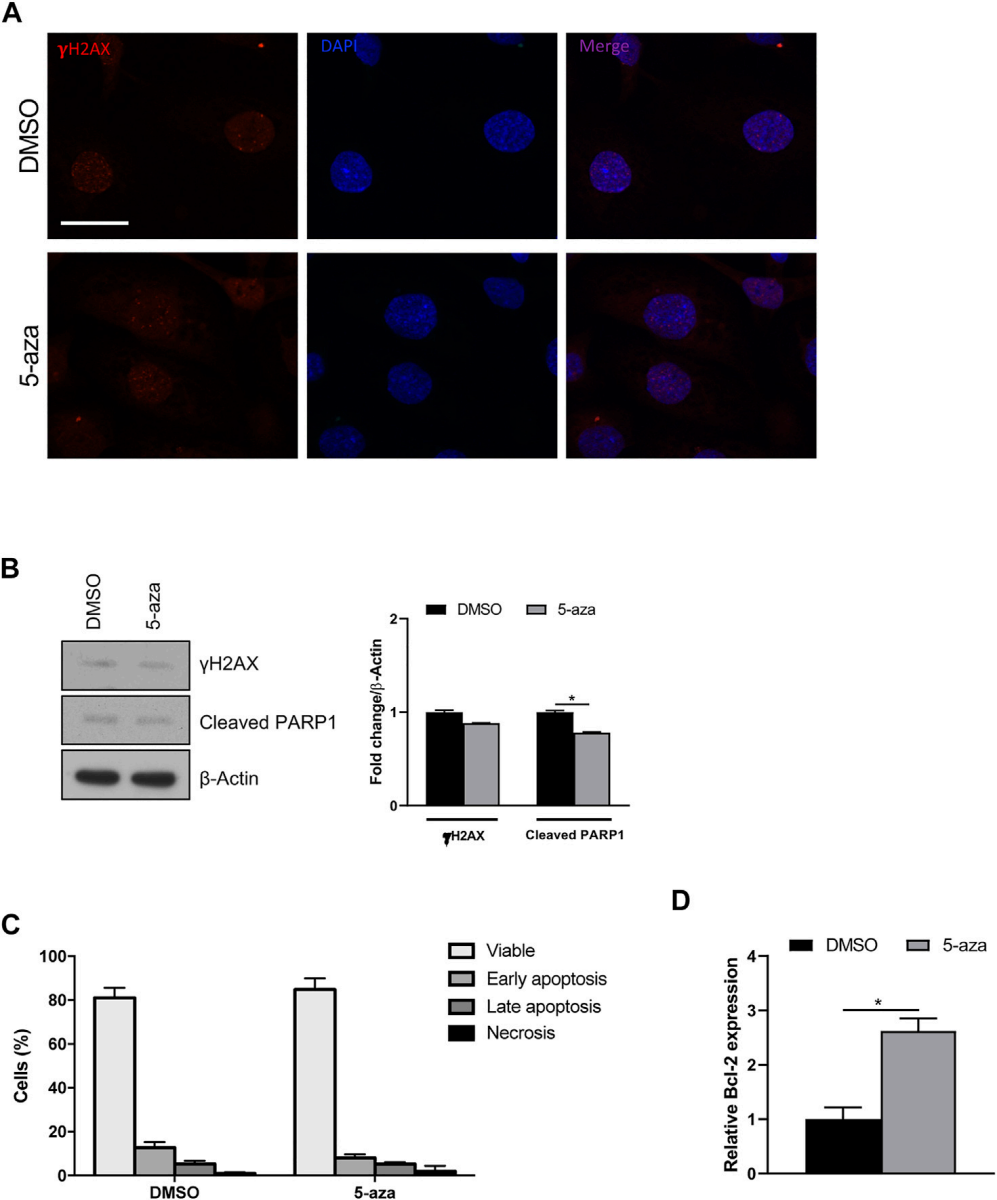
5-aza effects on DNA damage and apoptosis. **(A)** IF analysis showing γH2AX foci (red) in ASCs pretreated with 5-aza or DMSO. Nuclei were visualized using 4′, 6-diamido-2-phenylindole dihydrochloride (DAPI) (blue). Scale bar: 50 μm. **(B)** Western blot analysis of γH2AX and cleaved PARP1 protein expression. β-Actin was used as internal control. Densitometric analysis was reported as relative expression with respect to DMSO-treated cells. **(C)** Flow cytometry quadrant analysis with annexin A5 FITC/7-AAD double staining. The percentages of viable, early apoptotic, late apoptotic and necrotic cells are expressed as histograms. **(D)** Bcl-2 mRNA expression, evaluated by qRT-PCR. mRNA levels were normalized to GAPDH mRNA expression. Bars represent means ± SD of three independent experiments, each performed in triplicate. *p < 0.05 vs*.* DMSO.

Since it is known that senescence can be induced by a variety of stimuli, including DNA damage, we wondered if 5-aza pretreatment was able to increase DNA double-strand breaks in ASCs. IF analysis of histone H2A.X phosphorylation revealed that 5-aza pretreatment, at this dose and timing, does not induce an evident increase of γH2AX foci (Figure 8A). Such observation was further confirmed by WB analysis with γH2AX antibodies, showing no upregulation upon 5-aza treatment (0.9-fold; Figure 8B).

To determine whether 5-aza induces other cellular pathways than senescence, apoptosis induction was analyzed. WB analysis showed slightly decreased expression of cleaved PARP1, a known hallmark of caspase activation, upon 5-aza treatment (0.8-fold; Figure 8B). Also, flow cytometry quadrant analysis with annexin A5 FITC/7-AAD double staining showed that 5-aza treatment even reduced the percentage of apoptotic cells (18% in DMSO vs*.* 13.3% in 5-aza; Figure 8C). Accordingly, we further observed a significant increase in the mRNA expression of the anti-apoptotic factor Bcl-2 upon 5-aza treatment (2.6-fold; Figure 8D), which could be responsible for the determination of cellular senescence versus apoptosis. These data strongly suggested that 5-aza pretreatment induce a senescent phenotype rather than cell death in ASCs.

## Discussion

Soft tissue defects represent a complex challenge for the plastic surgeon, especially in the context of high prevalence of diseases such as diabetes mellitus, obesity, chronic venous insufficiency, and peripheral artery occlusive disease, or after radiation therapy (Wu et al., 2012). Autologous fat tissue transfer is commonly used for reconstructive purposes, but its efficacy is impaired by frequent implant loss, due to inadequate vascularization of the transplanted tissue, and volume loss, due to central necrosis of autologous adipose tissue (Rubin and Marra, 2013). So, clinical application of ASCs capable to *in vivo* differentiate into adipocytes could considerably ameliorate the outcome of reconstructive surgery, as long as issues concerning ASC survival, differentiation and migration capability in the transplanted microenvironment are resolved (Murry et al., 2004). In fact, suboptimal *in vivo* performance frequently occurs due to improper engraftment of applied cells, to be explained by both low cell survival and inefficient homing ability.

Stem cell differentiation involves epigenetic modifications of the cellular genome (Wu and Yi, 2006), and several studies reported an effect of DNA demethylating agents on this process. The treatment of the myoblast cell line C2C12 with the epigenetic modifier 5-aza has been observed to promote myogenic differentiation (Hupkes et al., 2011), whilst 5-azadeoxycytydine (5-azadC) is able to stimulate osteogenesis (El-Serafi et al., 2011) and to decrease adipogenesis in BM-MSC cultures, despite the induction of the adipogenic marker PPARγ immediately after treatment (Zych et al., 2013).

In particular, the nonspecific demethylation of DNA can modify gene activation and expression, this affecting multiple regulatory pathways (Komashko and Farnham, 2010).

In the present study, we investigated the effects of DNA demethylation on several aspects of ASC biology, including the senescence, proliferation, self-renewal and differentiation capacity, together with the migration potential. Indeed, we observed a detrimental effect of 5-aza on ASC clonogenicity and migration, and we analyzed the involvement of the Akt/mTOR pathway in these processes. Our results indicated an impairment of Akt phosphorylation, as well as a reduction of ERK activation, upon 5-aza pretreatment, this potentially explaining the reduced self-renewal and migration observed in functional assays.

The PI3K/Akt/mTOR axis is known to play a controversial role in adipogenesis. In fact, Yu et al. reported decreased adipogenesis upon inhibition of this pathway (Yu et al., 2008), while Fitter et al. showed that imatinib promoted adipogenesis by inhibiting PDGF-induced PI3K activity (Fitter et al., 2012). Our results indicate a significant enhancement of adipogenic differentiation by 5-aza, despite the reduction of Akt phosphorylation. Some reports indicated that activation of the downstream protein mTOR could be sufficient to promote adipogenesis through upregulation of the two master adipogenic transcriptional factors PPARγ and c/EBPα (Chen et al., 2013). Indeed, we demonstrated that 5-aza did not negatively affect mTOR phosphorylation and that upregulation of PPARγ mediated by 5-aza is strictly dependent on mTOR activation, since it is impaired in the presence of the mTOR inhibitor rapamycin. Also, MAPK pathway exerts both positive and negative roles in adipogenesis, since activation of ERK signaling is crucial to support the mitotic clonal expansion during the early phases but then its shut off is needed to allow adipogenic differentiation (Bost et al., 2005; Scioli et al., 2014). Previous works showed that activation of MAPK signaling can be responsible for the inhibition of adipogenesis, probably due to the ERK-mediated phosphorylation of PPARγ and the subsequent reduction of its transcriptional activity (Hu et al., 1996; Kim et al., 2007). So, the decreased ERK activity observed in our cells upon 5-aza treatment might further contribute to boost ASC adipogenesis.

Interestingly, the pro-adipogenic effect of 5-aza can be also explained by its ability to modify ASCs actin cytoskeleton, as demonstrated by IF with TRITC-phalloidin. Cells pretreated with 5-aza showed a reduction of actin stress fibers, which is compatible with reduced migration potential (Buchan et al., 2002; Kakinuma et al., 2008) and with increased ability to differentiate towards adipogenic lineage (Mathieu and Loboa, 2012; Chen et al., 2018). Also, the reduced number of focal adhesions observed by IF with Vinculin after 5-aza treatment is in line with previous report assessing that focal adhesion disruption increases the adipogenic potential of MSCs (Luo et al., 2008).

Since both mitogenic and differentiation signals might be induced by several signaling pathways, including that of Wnt/β-catenin, we also analyzed the effects of 5-aza on this axis, which is known to play a central role in stem cells differentiation. In particular, canonical Wnt pathway activation is essential to trigger mesenchymal stem cells differentiation towards osteogenic lineage (Kang et al., 2007), while it can be considered as a negative regulator of adipogenesis (Ross et al., 2000; Bennett et al., 2002), likely through indirect inhibition of the expression of the adipogenic regulators c/EBPα and PPARγ. Indeed, numerous studies have documented that pro-adipogenic factors might exert an antagonistic effect on this signaling pathway (de Winter and Nusse, 2021). Similarly, our results in the present study suggest that 5-aza treatment in ASCs may enhance adipogenesis through inhibition of the Wnt signal pathway, as indicated by the upregulation of the secreted Wnt inhibitor factor sFRP1 and prevention of β-catenin nuclear translocation, with subsequent downregulation of the constitutive Wnt targets Axin2, Cyclin D1 and c-Myc. A previous study showed that Axin2 downregulation might be responsible for the release of cytoplasmic Axin2-bound GSK3β, which nuclear translocation can activate the machinery for c/EBPα and PPARγ transcription (Xie et al., 2018). This is in line with our observations of 5-aza-mediated upregulation of these genes.

Such conclusions are further strengthened by reports assessing the ability of 5-aza to decrease β-catenin and cyclin D1 expression in endometrioid carcinoma and prostate cancer cells (Zhang et al., 2014; Liao et al., 2020). Furthermore, our data assessing 5-aza-induced Cyclin D1 downregulation and PPARγ stimulation are in agreement with the functional antagonism between these two molecules that has been demonstrated in fibroblasts (Wang et al., 2003).

It has been previously shown that Akt can phosphorylate β-catenin, thus promoting its transcriptional activity (Fang et al., 2007). In this light, the inhibition of Akt phosphorylation mediated by 5-aza could also contribute to the shutting off of Wnt/β-catenin signaling, highlighting a synergic positive effect of these two pathways on adipogenesis. Such conclusion is further strengthened by the ability of a combination of the mTOR inhibitor rapamycin and the Wnt activator Wnt3A to impair 5-aza-mediated enhancement of adipogenesis.

Treatment with 5-aza has been shown to induce senescence in solid tumors (Christman, 2002; Grandjenette et al., 2014; Putri et al., 2017). So, also considering the decreased Cyclin D1 expression levels upon 5-aza pretreatment, we hypothesize that DNA demethylation could trigger senescence also in ASCs. Our evidence of an increase in the number of senescent cells, as indicated by β-galactosidase staining, was further confirmed by upregulation of p21 and p53, which are known to be associated with cellular senescence in several cell types, including ASCs (Jain et al., 2012; Anastasiadou et al., 2021), and by the increased expression of senescence-associated secretory phenotype (SASP) components, namely SDF-1, MIF, PAI-1, IL-6 and IL-8 (Coppé et al., 2010). As for PAI-1, it is also known to activate the p53/p21 pathway (Jiang et al., 2017), so its upregulation by 5-aza is in accordance with the increase in p53 and p21 observed in 5-aza-treated cells. The downregulation of Lamin B1 observed in 5-aza pretreated cells is in line with the presence of senescent cells, which are often characterized by loss of nuclear integrity (Hernandez-Segura et al., 2018). We also assessed the potential of 5-aza to trigger DNA damage signals and execute apoptosis. The observation of no increased expression of γH2AX foci in 5-aza pretreated cells confirmed that our treatment is not sufficient to induce significant DNA strand breaks. Furthermore, both flow cytometry assay and WB for PARP1, a known cellular substrate of caspases whose cleavage is considered to be a hallmark of apoptosis (Kaufmann et al., 1993) revealed that the percentage of apoptotic cells is even reduced in response to 5-aza. It is known that the choice between cell survival and apoptosis after activation of p53 might depend upon the balance between expression of pro- and anti-apoptotic molecules, and that the anti-apoptotic factor Bcl-2 is important for cell survival during senescence (Rincheval et al., 2002). So, our observation of a Bcl-2 upregulation in 5-aza-treated cells strengthen the hypothesis that ASCs are induced to respond to p53 induction upon 5-aza treatment by entering a senescence state rather than activating cell death. Triggering of cellular senescence by 5-aza may ultimately be responsible of the detrimental effects on other important features of ASCs, such as cell self-renewal and migration, as observed in the colony formation and scratch/transwell assays.

Overall, our data showed that global demethylation induced by 5-aza pretreatment was able to enhance adipogenic potential of ASCs, but at the same time we found that pretreated cells partially lose their self-renewal capacity and accelerate their senescence program, this potentially impairing an efficient expansion process in culture. Further, 5-aza treatment could also impair ASC migration ability, which could have a negative impact on cell homing upon *in vivo* transfer. Such evidence clearly suggests that we need to be cautious with the use of demethylating strategies when designing ASCs clinical applications.

## Conclusion

**FIGURE 9:**
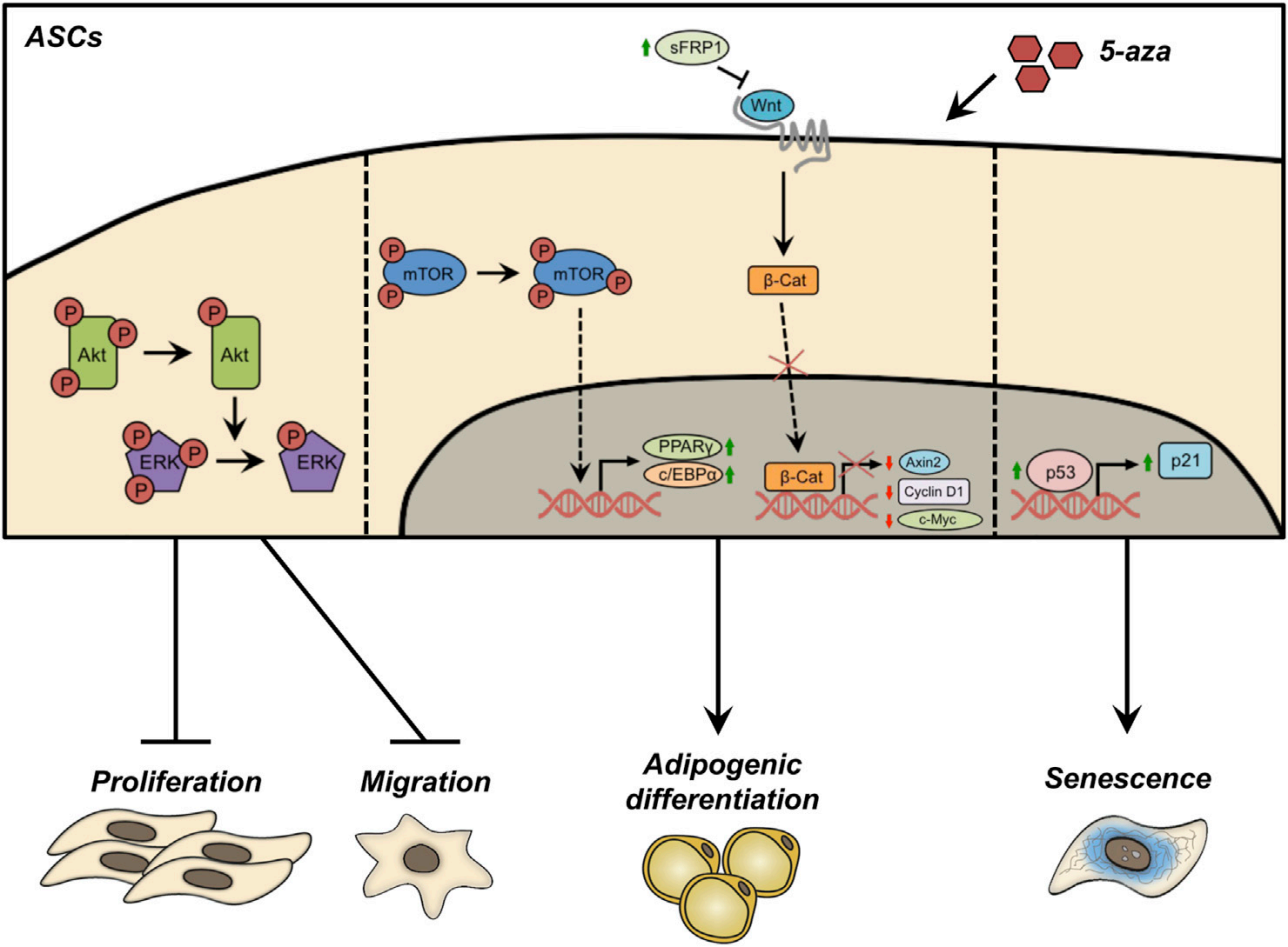
Schematic representation of the effects of 5-aza pretreatment on ASCs. Pretreatment with 5-aza inhibits the phosphorylation of Akt and ERK, reducing cell proliferation/migration. 5-aza treated ASCs maintained an Akt-independent activation of mTOR, allowing the activation of the master genes of adipogenesis, PPARγ and c/EBPα. The treatment with 5-aza also impairs the Wnt/β-catenin signaling, by increasing the expression of the Wnt inhibitor sFRP1, affecting β-catenin expression and nuclear translocation, and reducing the transcription of the Wnt target genes Axin2, Cyclin D1 and c-Myc. Finally, 5-aza pretreatment induces an upregulation of the p53/p21 axis and a decrease of Lamin B1 in the nuclear membrane, a known hallmark of cell senescence.

The present study investigates the effects of 5-aza pretreatment on proliferation, migration, adipogenic differentiation and senescence of human ASCs, which are summarized in Figure 9. The demethylation induced by 5-aza reduced cell proliferation/migration through inhibition of Akt and ERK phosphorylation. Conversely, 5-aza showed a positive effect on ASC adipogenic differentiation, potentially involving the Akt-independent activation of mTOR and/or the inhibition of Wnt/β-catenin signaling. Furthermore, 5-aza pretreatment also determined an increase in cell senescence, through upregulation of the p53/p21 axis. Our data have significant translational implications, allowing us to clarify the potential risks and advantages of using epigenetic approaches to improve ASC characteristics for cell-based clinical approaches.

## Data Availability

The original contributions presented in the study are included in the article/Supplementary Material, further inquiries can be directed to the corresponding author.
