# Supplementary material for: Inhibiting DNA methylation as a strategy to enhance adipose-derived stem cells differentiation: Focus on the role of Akt/mTOR and Wnt/β-catenin pathways on adipogenesis
Source: Front Cell Dev Biol. 2022 Sep 2;10:926180. doi: 10.3389/fcell.2022.926180 (PMC9478209; doi:10.3389/fcell.2022.926180)
Supplement: Supplementary file 1 [file DataSheet1.DOCX]

Supplementary Material

# Supplementary Materials and methods

## Cytokine array

ASC were seeded in a 100 mm dish and treated with DMSO or 5-aza for 48 h in growth media. The medium was replaced with fresh DMEM without growth factors. After 72 h, the remaining conditioned medium (CM) was harvested and tested for protein secretion by Human XL Cytokine Array Kit (Cat. No. ARY005B; R&D Systems). Membranes were treated and analyzed according to manufacturer protocol. Densitometric analyses were performed with Quantity One Program (Bio-Rad Laboratories S.r.l., Segrate, MI, Italy).

## Quantitative Real-Time PCR (qRT-PCR)

Total RNA was extracted and quantified as previously described. The abundance of specific mRNAs was quantified in triplicate on an ABI 7500 Real Time instrument (Applied Biosystems by Life Technologies) using the following TaqMan gene expression assay probes (Applied Biosystems by Life Technologies): PAI-1 (Hs00167155_m1) and IL-6 (Hs00174131_m1). GAPDH mRNA (Hs02758991_g1) was used as endogenous control.

# Supplementary Figures and Tables

## Supplementary Figures

**Supplementary Figure 1.** 5-aza induce a SASP phenotype in ASC. (A) Human cytokine array on the culture medium of ASCs pretreated with 5-aza or DMSO. Significantly changed cytokines in the presence of 5-aza pretreatment were marked and shown on the right. Black boxes represent positive control; black dotted boxes represent negative control. Densitometric analysis was reported as relative expression with respect to DMSO-treated cells. (B) IL-6 mRNA expression in ASCs pretreated with 5-aza or DMSO, evaluated by qRT-PCR. mRNA levels were normalized to GAPDH mRNA expression. Bars represent means ± SD of three independent experiments, each performed in triplicate. * p < 0.05 *vs* DMSO.
